# Pleiotropic effects in *Eya3 *knockout mice

**DOI:** 10.1186/1471-213X-8-118

**Published:** 2008-12-22

**Authors:** Torben Söker, Claudia Dalke, Oliver Puk, Thomas Floss, Lore Becker, Ines Bolle, Jack Favor, Wolfgang Hans, Sabine M Hölter, Marion Horsch, Magdalena Kallnik, Eva Kling, Corinna Moerth, Anja Schrewe, Christian Stigloher, Stefanie Topp, Valerie Gailus-Durner, Beatrix Naton, Johannes Beckers, Helmut Fuchs, Boris Ivandic, Thomas Klopstock, Holger Schulz, Eckhard Wolf, Wolfgang Wurst, Laure Bally-Cuif, Martin Hrabé de Angelis, Jochen Graw

**Affiliations:** 1 Helmholtz Center Munich, German Research Center for Environmental Health, Institute of Developmental Genetics, Neuherberg, Germany; 2 Helmholtz Center Munich, German Research Center for Environmental Health, Institute of Experimental Genetics, Neuherberg, Germany; 3 Helmholtz Center Munich, German Research Center for Environmental Health, Institute of Inhalation Biology, Neuherberg, Germany; 4 Department of Medicine III, Otto-Meyerhof-Center, Heidelberg, Germany; 5 Department of Neurology, Friedrich-Baur-Institut, Ludwig-Maximilians-University, Munich, Germany; 6 Institute of Molecular Animal Breeding and Biotechnology, Gene Center, Ludwig-Maximilians-University, Munich, Germany; 7 Technische Universität München, Center of Life and Food Sciences Weihenstephan, Chair of Experimental Genetics, Weihenstephan, Germany; 8 Technische Universität München, Center of Life and Food Sciences Weihenstephan, Chair of Developmental Genetics, Weihenstephan, Germany; 9 Present address: Fraunhofer IME, Aachen, Germany; 10 Present address: Max-Planck-Institut für Biochemie, Martinsried, Germany; 11 Present address: Ecole Normale Supérieure, Département de Biologie, INSERM U789, Paris, France; 12 Helmholtz Center Munich, German Research Center for Environmental Health, Institute of Human Genetics, Neuherberg, Germany

## Abstract

**Background:**

In *Drosophila*, mutations in the gene *eyes absent *(*eya*) lead to severe defects in eye development. The functions of its mammalian orthologs *Eya1-4 *are only partially understood and no mouse model exists for *Eya3*. Therefore, we characterized the phenotype of a new *Eya3 *knockout mouse mutant.

**Results:**

Expression analysis of *Eya3 *by *in-situ *hybridizations and β-Gal-staining of *Eya3 *mutant mice revealed abundant expression of the gene throughout development, e.g. in brain, eyes, heart, somites and limbs suggesting pleiotropic effects of the mutated gene. A similar complex expression pattern was observed also in zebrafish embryos.

The phenotype of young adult *Eya3 *mouse mutants was systematically analyzed within the German Mouse Clinic. There was no obvious defect in the eyes, ears and kidneys of *Eya3 *mutant mice. Homozygous mutants displayed decreased bone mineral content and shorter body length. In the lung, the tidal volume at rest was decreased, and electrocardiography showed increased JT- and PQ intervals as well as decreased QRS amplitude. Behavioral analysis of the mutants demonstrated a mild increase in exploratory behavior, but decreased locomotor activity and reduced muscle strength. Analysis of differential gene expression revealed 110 regulated genes in heart and brain. Using real-time PCR, we confirmed *Nup155 *being down regulated in both organs.

**Conclusion:**

The loss of *Eya3 *in the mouse has no apparent effect on eye development. The wide-spread expression of *Eya3 *in mouse and zebrafish embryos is in contrast to the restricted expression pattern in *Xenopus *embryos. The loss of *Eya3 *in mice leads to a broad spectrum of minor physiological changes. Among them, the mutant mice move less than the wild-type mice and, together with the effects on respiratory, muscle and heart function, the mutation might lead to more severe effects when the mice become older. Therefore, future investigations of *Eya3 *function should focus on aging mice.

## Background

*Eya3 *is one of four mammalian orthologous genes (*Eya1-4*) of *eyes absent *(*eya*) in *Drosophila melanogaster *[1,2]. Previous investigations demonstrated that a homozygous knockout of *eya *function in *D. melanogaster *results in severe embryonic defects and absence of compound eyes due to eye progenitor cell death [3,4]. Like *eyes absent *in *Drosophila*, mammalian *Eya *genes encode for transcriptional co-activator proteins and are widely co-expressed with *Pax6*, *Six1*, *Six2 *and *Dachshund (Dach) *genes. It is assumed that *Eya *genes are part of a hierarchically organized and highly conserved regulatory network during eye development with *Pax6 *on its top and *Six *and *Dach *genes as major players [5].

Eya proteins interact as transcriptional cofactors with Six and Dachshund proteins and do not possess DNA-binding properties. Eya proteins are characterized by a conserved 271-aa C-terminal domain (Eya domain, ED) which is believed to have a dual function [6]: on one hand, it mediates protein-protein interactions with Six and Dachshund proteins [7,8]; it is further required for nuclear translocation of Eya proteins [9]. On the other hand, *in-vitro *experiments demonstrated a tyrosine-phosphatase activity of the ED suggesting a catalytic function of this domain [10,11]. A second and far less conserved domain is the Eya domain 2 (ED2), which is located in the N-terminal region of the protein and presumably serves as a transactivation domain [12].

The relevance of mutations in the ED for the emergence of diseases has been widely shown for *Eya1*. In humans, *EYA1 *haploinsufficiency is responsible for branchio-oto-renal (BOR) syndrome or branchio-oto (BO) syndrome. BOR is an autosomal dominant disorder characterized by branchial cysts, ear malformations, hearing loss and renal abnormalities. Interestingly, the majority of BOR disease-associated missense mutations clusters in the ED and therefore suggest an important role for the ED in the development of these diseases [13]. Mice in which 153 of 271 amino acids of the Eya1-ED have been deleted die at birth and show severe craniofacial and skeletal defects and an absence of ears, kidneys, thymus and parathyroid glands [14]. *Eya1*^+/- ^mice show a phenotype, which is comparable to humans suffering from BOR syndrome. They display renal abnormalities and conductive hearing loss. Neither *Eya1*^+/- ^nor *Eya1*^-/- ^mice reveal ocular defects and only in a few cases could *Eya1 *mutations be associated with congenital cataracts and ocular anterior segment anomalies in humans [15].

Moreover, *Eya *genes are associated with the proper development of muscles. *Drosophila eya *mutants show musculature defects [16]; mouse embryos deficient for *Eya1 *and *Six1 *have a complete absence of all hypaxial muscle and a severe reduction of epaxial muscle [17]. While neither in *Eya1*^-/- ^nor in *Eya2*^-/- ^mice can a muscle phenotype be observed, *Eya1*^-/-^*Eya2*^-/- ^double knockout embryos display muscle-less limbs [18]. For *Eya2 *it was shown in tissue culture experiments that it interacts with *Six1 *and *Dach2 *to regulate *Pax3 *and therefore to influence myogenic differentiation [19]. In addition, *Eya *genes seem to play a role in heart and auditory function in vertebrates. Mutations of the *EYA4 *gene resulting in truncated EYA4 proteins cause dilated cardiomyopathy and conductive hearing loss in humans [20,21] and *Eya4 *mutations in mice are responsible for an abnormal morphology of the middle ear [22]. Injection of morpholino oligonucleotides against *Eya4 *into zebrafish (*Danio rerio*) embryos indicated an abnormal morphological and physiological phenotype of the heart [20]. Obviously, *Eya *genes are involved in the proper development of many tissues in vertebrates including the ear, muscles, heart, renal system and possibly the eye.

However, the influence of *Eya3 *on developmental processes in mice and other mammals is rather unknown. *In-vitro *experiments showed that *Eya3 *is important for cell-autonomous proliferation of murine myoblast C2C12 cells [17]. In addition, studies in *Xenopus *revealed a strong influence of the *Eya3 *homologous gene *Xeya3 *on survival and proliferation of neural progenitor cells in the anterior neural plate of *Xenopus *embryos [23]. There are, however, no indications for a similar expression pattern, neither in fishes nor in mice, and we cannot observe a brain phenotype in our mouse *Eya3 *mutants. To our knowledge, this *Eya3*-deficient mutant is the first mouse model for a mutation affecting *Eya3 *and therefore an excellent model to study its function in mammals. Our systemic analysis within the German Mouse Clinic (GMC) demonstrated a broad spectrum of minor physiological changes in the *Eya3*-deficient mice. Among them, the reduced movement of the mutant mice and effects on their respiratory, muscle and heart function are the most striking changes.

## Results

### 1. Molecular characterization of *Eya3*-deficient mice

**Figure 1:**
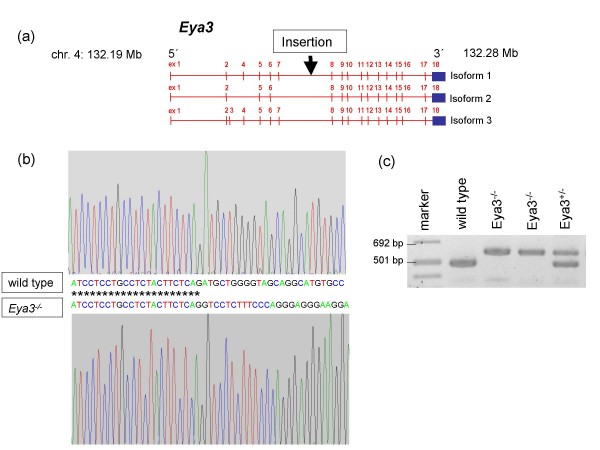
**Generation of *Eya3*-deficient mice by insertional mutagenesis**. (a) Schematic illustration of the gene trap vector integration into intron 7 of the *Eya3 *gene resulting in a loss of the following exons. (b) Sequence fragment of the *Eya3 *gene. The upper chromatogram displays the sequence of wild-type mice, while the lower chromatogram shows the vector integration in mutant mice at 132.23 Mb on chromosome 4 (*Ensembl *database, release No. 50, 2008). (c) *Eya3 *mutant mice were genotyped by triplex PCR with a genomic forward primer combined with a genomic reverse primer for identification of wild-type animals and with a reverse primer within the gene trap vector for identification of homozygous mutant animals.

Gene trap vector insertion was detected in a 16.7 kb large intronic region of the *Eya3 *gene (intron 7) using 5'RACE and PCR experiments and combining different forward primers and a reverse primer in the *lac*Z cassette of the pT1βgeo-vector. By cloning and sequencing of a 2.1 kb fragment in this region the integration site was determined to 132.23 Mb on chromosome 4 (*Ensembl *database, release No. 50, 2008) (Fig. 1a, 1b). Genotyping of mice confirmed the vector integration at the determined locus (Fig. 1c).

**Figure 2:**
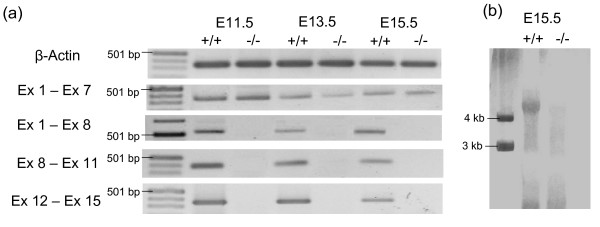
**Mutation of *Eya3 *causes a loss of exons 8 – 15 in homozygous mutants**. (a) RT-PCR with cDNA of wild type and *Eya3*^-/- ^embryos at developmental stages E11.5 (whole embryo), E13.5 (head), E15.5 (head) showed the loss of exons 8 – 15 of the *Eya3 *gene in *Eya3*^-/- ^mice. (b) Northern-Blotting using RNA of wild-type and *Eya3*^-/- ^embryos (E15.5) with a riboprobe against exons 8 – 11 confirmed the data received from RT-PCR.

RT-PCR and Northern-Blotting demonstrated the complete loss of exons 8–15 of the *Eya3 *gene in homozygous mutant animals. Transcripts containing exons 1–8 were detected in wild-type and heterozygous embryos at the age of E11.5 (whole embryo), E13.5 (head) and E15.5 (head). In contrast, no transcripts covering the exons 1–8, 8–11 or 12–15 were detectable in homozygous *Eya3*-mutants (Fig. 2a). Additionally, using a probe covering exons 8–11, Northern-Blotting (RNA from E15.5) were consistent with the results received from RT-PCR (Fig. 2b).

150 offspring from heterozygous matings have been checked: there were 76 males (50.5%) and 74 females (49.5%); 35 animals were wild type (23%), 85 heterozygous (57%) and 30 animals homozygous mutants (20%); the mean litter size was 5.2 offspring. These date indicate no major deviation from the expected numbers according to the Mendelian laws suggesting normal fertility of the heterozygotes and survival of the homozygous mutants.

### 2. Expression pattern of *Eya3 *in the mouse and consequences of *Eya3 *invalidation

**Figure 3:**
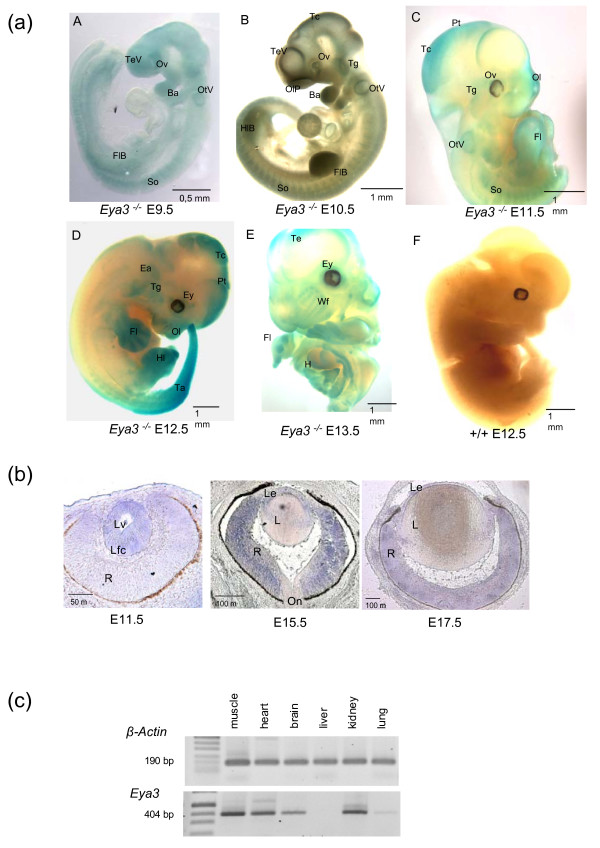
**Expression pattern of *Eya3 *in mice**. (a) Expression analysis of *Eya3 *by *lac*Z-staining in homozygous *Eya3*^-/- ^embryos (A – E, E9.5 – E13.5) revealed an expression of *Eya3 *in specific regions including the eyes, limbs, somites, branchial arches, tectorial regions, and in different areas of the developing brain. Since the expression pattern of *Eya3 *is identical, if analyzed by *in-situ *hybridization in wild-type embryos or by β-Gal expression in *Eya3*^-/- ^mutant mice, only the mutant data are shown. Wild-type embryos as negative controls did not show *lacZ*-staining (F). (Ba, branchial arches; Ey, eye; Ea, ear; Fl, forelimb; FlB, forelimb bud; H, heart; Hl, hindlimb; HlB, hindlimb bud; OlP, olfactory pit; Ol, olfactory region; Ov, optic vesicle; OtV, otic vesicle; Pt, pretectorial region; So, somites; Tc, tectorial region; Ta, tail; Te, telencephalon; TeV, telencephalic vesicle; Tg, trigeminal nerve; Wf, follicle of whiskers). (b) Expression analysis of *Eya3 *in the eye with *in-situ *hybridization displayed transcripts in the detaching lens at E11.5 and afterwards in the lens epithelium and in the retina (E15.5 – E17.5) (L, lens; Le, lens epithelium; Lfc, lens fiber cells; Lv lens vesicle; R, retina; On, optic nerve). (c) RT-PCR with cDNA of adult mice showed an *Eya3 *expression in muscle, heart, brain, kidney and lung, however, no *Eya3 *transcript was found in the liver.

*Eya3 *shows a complex expression pattern during embryonic development. The expression pattern of *Eya3 *is identical, if analyzed by *in-situ *hybridization in wild-type embryos or by β-Gal expression in *Eya3*^-/- ^mutant mice; data gathered from β-Gal staining of mutant embryos (Fig. 3a) demonstrated an expression of *Eya3 *at E9.5 in the developing neural tissues predominantly in the neural epithelium of the telencephalic vesicle and the later forebrain. Further on, *lac*Z staining is found in the somites, in the mandibular and maxillary component of the first branchial arch, in the optic vesicle and in the otic vesicle. In addition, an expression in the forelimb bud is visible. The expression of *lacZ *continues at the developmental stage E10.5 in the epithelium of the telencephalic vesicle and in tectorial regions of the developing brain. Moreover, *Eya3 *expression is indicated in the region surrounding the olfactory pit, the somites, fore- and hindlimb bud and in the trigeminal nerve. The first and second branchial arches display *Eya3/lac*Z transcripts, but the expression in the first branchial arch is mainly restricted to the mandibular component. At E11.5 *Eya3 *expression is detectable in the olfactory pit, the developing eye, the otic vesicle, tectorial and pretectorial regions of the brain, fore- and hindlimbs and in somites. At E12.5, *Eya3 *transcripts are most prominent in the limbs, in the tail and in cortical regions of the brain. Later (E13.5), the expression of *Eya3 *continues in the cerebellar cortex and in the limbs as well as in the ventral and dorsal grey horn of the spinal cord (not shown).

*Eya3 *expression in the eye (Fig. 3b) is first visible at E10.5 in the detaching lens vesicle. Later on transcripts are restricted to the retina and the lens epithelium (E15.5 – E17.5). In adult mice (Fig. 3c), an expression of *Eya3 *was traceable by RT-PCR in the brain, skeletal muscles, heart and kidneys. The lung displayed only a very weak *Eya3 *expression, while in the liver no expression was detected.

### 3. *Eya3 *expression in zebrafish development

**Figure 4:**
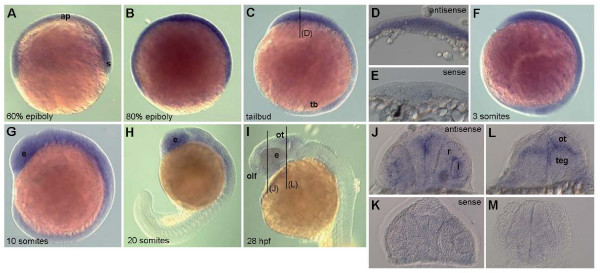
**Whole-mount *in-situ *hybridization for *eya3 *expression in zebrafish embryos**. Whole-mount *in-situ *hybridization for *eya3 *expression in zebrafish embryos reveals a ubiquitous expression of *eya3 *throughout gastrulation and early somitogenesis (A-F). Cross sections at the tailbud stage (level indicated in C) confirm expression in all cell layers (D), compared to a hybridization using the sense probe (E). The expression is more restricted to the anterior part of the embryo at 14 hpf (G). From 19 hpf the expression is most prominent in the developing brain and eye (H). By the beginning of the second day (28 hpf) (I) *eya3 *expression strongly decreases and is only weakly detectable in the eye, optic tectum, tegmentum and olfactory placode. This is confirmed on cross sections (J, L, at the levels indicated in I) compared to hybridization with the sense probe (K, M). Abbreviations: ap, animal pole; e, eye; l, lens; ot, optic tectum; olf, olfactory placode; r, retina; s, shield; tb, tailbud; teg, tegmentum.

Because of the obvious strong difference in the *Eya3 *expression pattern between mouse and *Xenopus*, we investigated *Eya3 *expression also in zebrafish embryos. Like at early developmental stages in mice, whole mount *in-situ *hybridization revealed a ubiquitous expression of *eya3 *in early zebrafish embryos throughout gastrulation and early somitogenesis. At the 10-somite-stage (14 hours post-fertilization, hpf), expression becomes more restricted to the anterior part of the embryo and at the 20-somite-stage (19 hpf) it is most prominent in the brain and developing eye. By the beginning of the second day of development (28 hpf), *eya3 *expression strongly decreases and becomes only weakly detectable in the eye, optic tectum and olfactory placode (Fig. 4).

### 4. General characterization of the *Eya3*^-/- ^phenotype

In cooperation with the German Mouse Clinic [24] 24 wild-type controls (14 ♂, 10 ♀) and 24 *Eya3*^-/- ^mice (14 ♂, 10 ♀) have been analyzed in a comprehensive phenotypic screen.

*Eya3 *mutant animals have been investigated for bone- and weight-related quantitative parameters at the age of 18 weeks. Among these parameters, the homozygous *Eya3 *mutants showed significant differences in bone mineral content, body weight and body length as compared to the control littermates. At the age of 18 weeks, these three parameters were significantly (p < 0.05) reduced in male mutants. Female mutants also displayed lower values compared to wild-type animals without reaching the level of statistical significance (table 1).

**Table 1:** Bone- and weight-related quantitative parameters (18 week old mice)

	**Wild type**		***Eya3*^-/-^**	
	male	female	male	female
Parameter	n = 9	n = 9	n = 10	n = 9
Bone mineral content [mg]	557 ± 33	467 ± 21	422 ± 39*	447 ± 33
Body length [cm]	9.06 ± 0.06	8.89 ± 0.07	8.60 ± 0.15*	8.67 ± 0.08
Body weight [g]	27.29 ± 0.63	21.05 ± 0.44	23.96 ± 1.12*	20.57 ± 0.82

*p < 0.05 (as compared to the corresponding group of wild-type mice); data are presented as mean ± standard error of the mean

**Figure 5:**
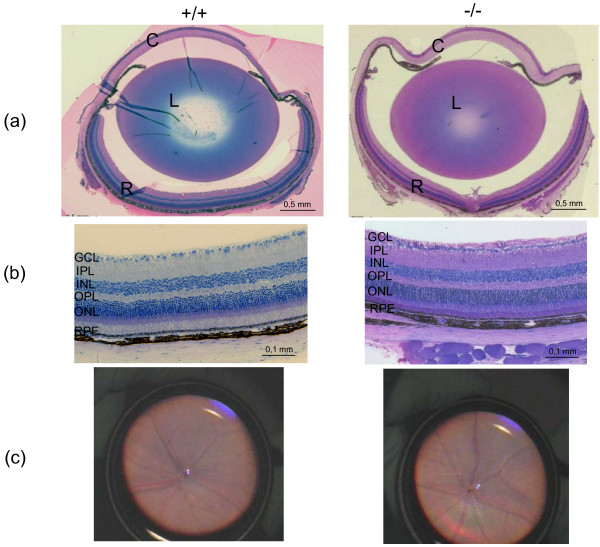
**Histology and funduscopy of the eye in *Eya3 *mouse mutants**. (a) Histology of the eye showed no morphological differences between wild-type mice and homozygous mutants (C, cornea; L, lens; R, retina). In line with these results histological analysis (b) and funduscopy (c) of the retina could not detect structural impairments in mutant animals (GCL, ganglion cell layer; IPL, inner plexiform layer; INL, inner nuclear layer; OPL, outer plexiform layer; ONL, outer nuclear layer; RPE retinal pigment epithelium).

**Figure 6:**
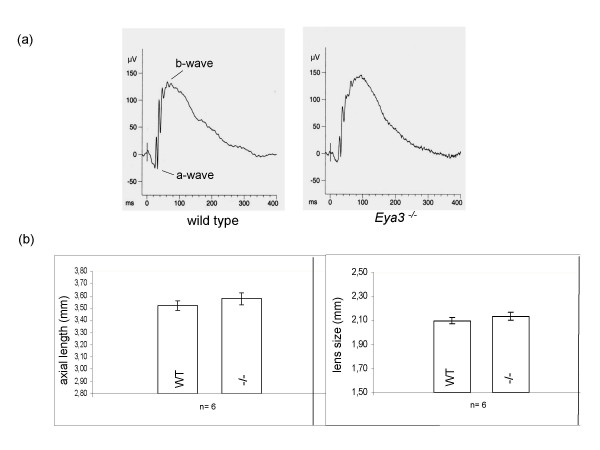
**Electroretinography (ERG) and laser interference biometry**. (a) The electroretinography displays no obvious deviations in the shape of the a-wave (response of photoreceptor cells) and b-wave (response of bi-polar cells) between wild-type controls and *Eya3 *mutant animals. (b) Measurement of the axial length of the entire eye and of the lens size in particular did not reveal significant alterations in *Eya3 *mutant animals compared to wild-type controls.

Data from expression analysis demonstrated that *Eya3 *is expressed in the developing ear and in the adult kidney. However, no significant differences between *Eya3 *knockout and wild-type animals have been observed in either functional or histological analyses, suggesting that *Eya3 *is not a major player in these organs. Moreover, expression analysis displayed that *Eya3 *is expressed in the retina and in the lens epithelium of the eye (Fig. 3b), suggesting that *Eya3 *may be important for proper lens and retina development. However, **histological analysis **of eyes taken from adult mice revealed no morphological differences between wild-type controls and homozygous *Eya3 *mutant mice (Fig. 5a, b). Further on, the investigation of the posterior part of the eye by **funduscopy **(Fig. 5c) did not show striking phenotypical differences of the retinal structure between wild-type and mutant animals. In addition to the morphological examination of the retina a rough functional analysis was done by **electroretinography **(ERG) (Fig. 6a). Measurement of retinal and ganglion cell response to the light stimulus displayed no significant (p > 0.05) deviations in mutant animals compared to wild-type controls.

An investigation of the eye size by **laser interference biometry **was performed to identify eye size variation between wild-type controls and *Eya3 *mutant animals (Fig. 6b). Anterior chamber depth, lens size and axial length were measured revealing no significant (p > 0.05) deviations between wild-type mice and homozygous *Eya3 *mutant mice.

#### Alteration in lung, heart and muscle function

The expression analysis (Fig. 3) demonstrated that *Eya3 *is expressed in heart and muscle, but only weakly in the lung. Nevertheless, systematic analysis of the function of these three organ systems showed significant alterations.

To evaluate the **lung function**, several parameters have been tested in male *Eya3*^-/- ^mutants and compared to wild-type littermates (tidal volumes, respiratory rates, minute ventilation, inspiratory and expiratory times as well as peak inspiratory and peak expiratory flow rates). Among these parameters, the tidal volume at rest is significantly reduced (p < 0.05) in the mutants (+/+: 0.25 ± 0.01 ml, n = 5; *Eya3*^-/-^: 0.22 ± 0.01 ml, n = 4). This difference is more striking (p < 0.02), if related to the body weight (+/+: 10.1 ± 0.3 μl/g, n = 5; *Eya3*^-/-^: 8.7 ± 0.3 μl/g, n = 4). In this context, it might be of interest that the respiratory rate of the mutant mice is enhanced, even if it does not reach the level of statistical significance (+/+: 302.2 ± 9.3/min, n = 5; *Eya3*^-/-^: 342.6 ± 19.1/min, n = 4). However, this may suggest that compensatory mechanisms are triggered. The reduced muscle strength – if transferable to respiratory muscles – may limit the potential volume to be inspired and, thus, result in the observed reduced tidal volume. Consequently, the respiratory rate is increased leading to strikingly similar minute ventilations in wild type and *Eya3*^-/- ^(72.7 ± 3.5 ml/min vs. 71.0 ± 1.6 ml/min).

**Figure 7:**
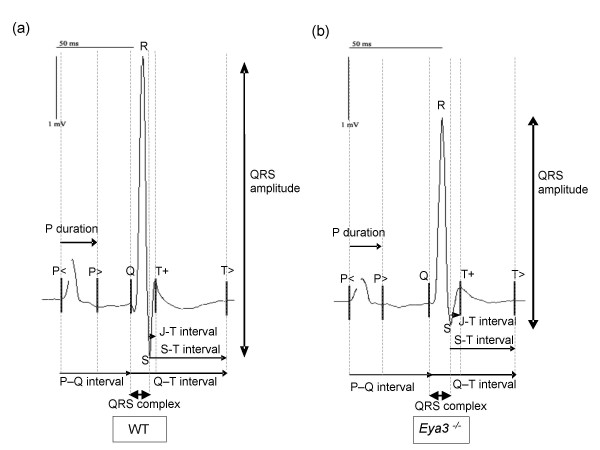
**Electrocardiography (ECG) of wild-type and *Eya3*^-/- ^mutant mice**. a) Electrocardiogram of a wild-type control. b) Electrocardiogram of an *Eya3*^-/-^animal is shown. *Eya3 *mutant mice of both sexes displayed several alterations compared to the control animals: the PQ-interval and the JT-interval are extended in mutants, while the amplitude of the QRS-complex is reduced in *Eya3*^-/- ^animals (alterations are indicated by black arrows).

In addition, **cardiovascular function **was examined by tail-cuff blood pressure and by electrocardiography (ECG). Analysis of blood pressure revealed no genotype-specific differences between wild-type and mutant mice. In contrast, the ECG showed significant genotype-specific differences between controls and mutants of both sexes (Fig. 7). The time from onset of the electrical impulse in the sinu-atrial node until ventricular depolarization (PQ-interval) was increased. Additionally, the time from ventricular depolarization to repolarization (JT-interval) was extended, and the amplitude of the QRS-complex representing ventricular depolarization was highly significantly decreased in mutant animals (table 2).

**Table 2:** ECG-Parameters (15 week old mice)

	**Wild type**		***Eya3*^-/-^**	
	male	female	male	female
Parameter	n = 8	n = 9	n = 10	n = 9
PQ interval [ms]	38.6 ± 0.7	40.8 ± 0.7	41.3 ± 1.6*	43.6 ± 0.9*
P-wave duration [ms]	20.4 ± 0.6	20.4 ± 0.8	20.8 ± 0.6	21.4 ± 0.3
QRS-complex duration [ms]	9.6 ± 0.2	9.6 ± 0.2	10.0 ± 0.2	9.8 ± 0.3
QT interval [ms]	46.2 ± 1.3	41.7 ± 0.6	45.6 ± 2.1	42.7 ± 0.6
QT_corrected _[ms]	40.6 ± 1.4	36.5 ± 0.5	40.1 ± 1.5	38.2 ± 0.7
RR interval [ms]	130.6 ± 3.8	130.9 ± 4.1	130.1 ± 4.9	125.2 ± 3.7
Heart rate [bpm]	463.7 ± 13.5	463.4 ± 14.2	469.1 ± 18.6	482.7 ± 13.3
JT interval [ms]	4.1 ± 0.6	3.7 ± 0.1	6.0 ± 1.1*	5.4 ± 0.4*
ST interval [ms]	36.6 ± 1.5	32.1 ± 0.6	35.6 ± 2.1	32.9 ± 0.6
Q amplitude [mV]	0.01 ± 0.00	0.02 ± 0.01	0.04 ± 0.01**	0.03 ± 0.01**
R amplitude [mV]	3.10 ± 0.18	3.16 ± 0.35	2.32 ± 0.14**	2.33 ± 0.29**
S amplitude [mV]	-1.26 ± 0.22	-1.03 ± 0.16	-0.95 ± 0.14	-1.01 ± 0.16
QRS amplitude [mV]	4.35 ± 0.17	4.19 ± 0.40	3.28 ± 0.13***	3.34 ± 0.22***

*p < 0.05; **p < 0.01; ***p < 0.001 (as compared to the corresponding group of wild-type mice); data are presented as mean ± standard error of the mean

For evaluation of the **behavioral phenotype**, the spontaneous activity of mice was measured using a modified hole-board test. The mutants demonstrated a mild increase in exploratory behavior as indicated by a reduced latency to hole exploration. The frequency of rearings in the box was also enhanced in the mutants. However, the difference did not reach the level of statistical significance. In contrast, mutant mice exhibited a reduced forward locomotor activity, measured as reduced distance travel, reduced maximum velocity and reduced frequency of turns; the mean velocity was also reduced, but the difference did not reach the level of significance (table 3). The reduced locomotor activity might be associated with reduced muscle strength in these mice as indicated by the results of the forelimb grip strength test: *Eya3 *mutant mice of both sexes revealed a significant (p < 0.05) reduction of muscle strength. However, the performance on the Rotarod was only slightly impaired and did not reach the level of statistical significance.

**Table 3:** Results of behavioural observations (8–10 week old mice).

	**Wild type**		***Eya3*^-/-^**	
	male	female	male	female
Parameter	n = 15	n = 9	n = 15	n = 9
Forward locomotor activity (total distance travelled) [cm]	3387.2 ± 124.9	3534.3 ± 115.5	3086.8 ± 126.5*	3248.6 ± 71.4*
Speed of movement (maximum velocity) [cm/sec]	62.9 ± 1.9	59.8 ± 3.2	55.0 ± 1.9*	58.2 ± 1.9*
Turns [frequency]	1714.9 ± 46.1	1776.8 ± 41.7	1610.3 ± 41.6*	1688.8 ± 39.8*
Exploratory behaviour (hole exploration) [latency]	14.6 ± 3.7	13.9 ± 3.1	8.1 ± 1.5*	8.5 ± 1.6*
Grip strength (force) [p]	121.7 ± 2.8	99.3 ± 3.7	102.8 ± 4,9**	89.9 ± 3.9**

*p < 0.05; **p < 0.01 (as compared to the corresponding group of wild-type mice); data are presented as mean ± standard error of the mean

#### Analysis of differentially expressed genes in *Eya3 *mutant mice

**Figure 8:**
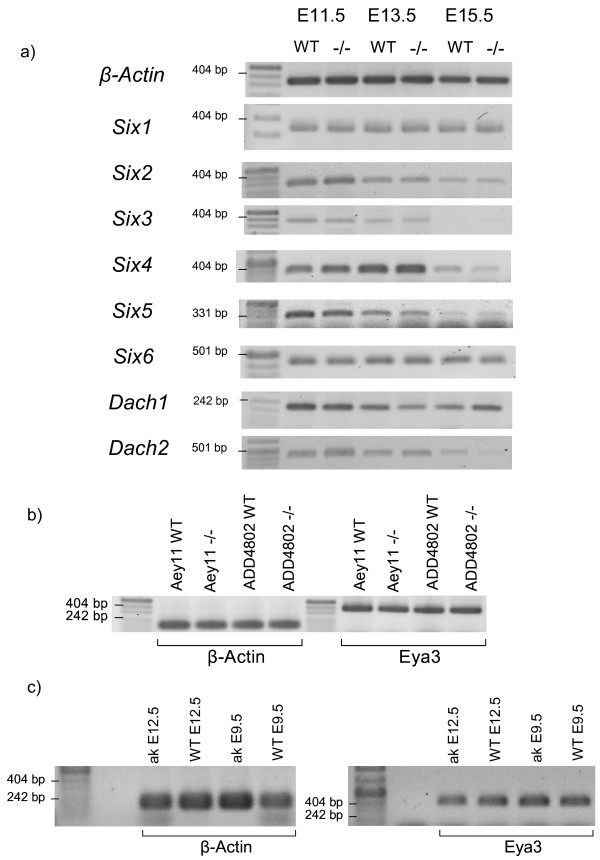
***Eya3 *and the retinal determination network**. a) The expression of all six *Six *genes (*Six1 *– *Six6*) and the two *Dach *(*Dach1*, *Dach2*) genes have been investigated in wild-type and homozygous mutant embryos by RT-PCR using total mRNA; β-actin was used as loading control. b) *Eya3 *expression was tested in embryos of two homozygous *Pax6 *mutant alleles (*Pax6*^*Aey*11 ^and *Pax6*^*ADD*4802^); β-actin was used as loading control. b) *Eya3 *expression was tested in embryos of a homozygous *Pitx3 *mutant allele (*Pitx3*^*ak*^); β-actin was used as loading control.

Since the members of the *Eya *gene family are discussed to be members of a conserved regulatory network during eye development including also *Six *and *Dach *genes and *Pax6 *on its top [5], we tested the influence of the loss of *Eya3 *activity on their expression. Fig. 8a demonstrates that all six *Six *genes as well as the two *Dach *genes are not changed in their overall expression at least within the critical phase of early eye development between E11.5 and E15.5. On the other side, we checked also the *Eya3 *expression in two *Pax6 *mutant alleles (Fig. 8b; allele symbols *Aey11*, *ADD4802*; 39); the overall *Eya3 *expression is obviously not altered even in homozygous *Pax6 *mutant embryos. The same is true in a *Pitx3 *mutant *aphakia *(allele symbol *ak*; Fig. 8c; [25]). These results indicate that Eya3 is no major player in the regulatory network of eye development.

**Figure 9:**
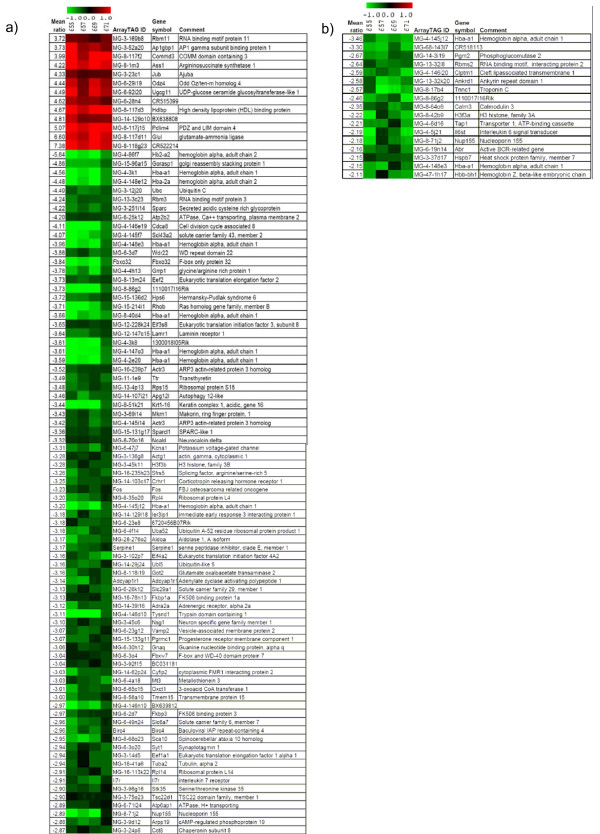
**Heat plots of gene expression profiles from 8 DNA microarray experiments of *Eya3 *mutants versus control mice**. Differential gene expression was analyzed in the brain (a) and in the heart (b). One dye-flip pair represents two experimental replicates of each analyzed mouse (A-D). One ArrayTAG ID is the unique probe identifier from the LION Bioscience clone set. Official gene symbols are given. The scale bar encodes the ratio of the fold induction; 0.7% (a) or 1.3% (b) of the elements are above the upper limit of the color range selection (red is up-regulated and green down-regulated in the *Eya3 *mutant mice).

To obtain a systematic overview on the expression of downstream target genes, we performed an expression profiling analysis using a genome-wide cDNA microarray approach. Gene expression patterns of skeletal muscle, heart, and brain of adult, homozygous *Eya3 *mutants were compared to wild-type littermates. In skeletal muscles of *Eya3 *mutant mice no significantly regulated genes were detected between mutant and wild-type tissue. In contrast, 93 differentially expressed genes were identified in brain (13 up- and 80 down-regulated genes; Fig. 9a). In heart tissue all 17 differentially regulated genes showed decreased expression levels in the *Eya3 *mutant mice (Fig. 9b). Due to the limited number of regulated genes, no over-representation of Gene Ontology (GO) terms could be identified in these 17 genes. However, among the differentially regulated genes in the brain, the macromolecule biosynthetic process was over-represented for biological processes, and the structural constituent of cytoskeleton and structural constituent of ribosome were over-represented for molecular functions.

Among these differentially regulated genes, *Nup155 *(*Nucleoporin 155*) was significantly down-regulated both, in heart (2.1-fold) **and **brain (2.8-fold). Validation of *Nup155 *expression level with real-time PCR confirmed the differential regulation in both organs of the *Eya3*^-/- ^mutant mice (heart: 1.6-fold, p < 0.05; brain: 2.2-fold, p < 0.01). Since the Nup155 protein is discussed to be involved in the transport of proteins and mRNA, its reduced expression in brain and heart might also contribute to the alterations observed in the behavioral phenotype of the *Eya3 *mutant mice and of their slightly altered cardiac function.

## Discussion

The gene *eyes absent *was shown to have a prominent function during eye development of *Drosophila melanogaster *[3]. In contrast, the mammalian orthologs seem to play a far less important role in eye development.

In contrast to *Xenopus *embryos, where the expression of *Xeya3 *is restricted exclusively to the anterior neural plate and cells of the prospective brain and eye within neurulation (Kriebel et al., 2007), our results from mice and zebrafish display a ubiquitous expression of *Eya3 *in early embryos during neural plate stage. It is only by the end of neurulation that a restriction of *Eya3 *expression to distinct regions like forebrain and eye in mice or optic tectum and eye in zebrafish is observable. Thus, our expression analysis for *Eya3 *in mouse and zebrafish demonstrates striking differences between these organisms and *Xenopus*, suggesting the possibility of different functions for *Eya3 *among vertebrate species.

**Figure 10:**
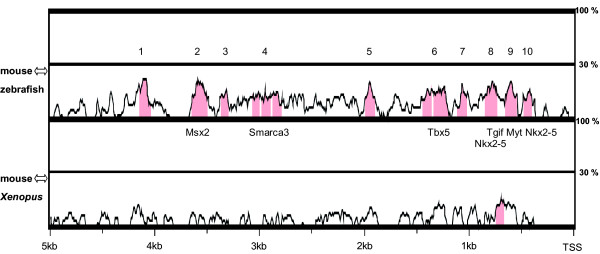
**Conserved elements in *Eya3 *promoter/enhancer region**. Sequence analysis of 5 kb upstream from *Eya3 *transcriptional start site for mouse, zebrafish and *Xenopus *using mVISTA (parameters: 50% sequence identity, 80 bp window length). The analysis revealed 10 conserved non-coding regions within the mouse-zebrafish alignment (sequence identity: 1325 bp at 52.9%, highlighted in red) and only 1 conserved non-coding region for the mouse-*Xenopus *alignment (sequence identity: 78 bp at 50%). Transcription factor binding sites of a matrix similarity of 1.000 are mentioned. TSS, transcription start site.

This might be due to differences in regulatory mechanisms of *Eya3 *between these organisms. Comparative sequence analysis of the *Eya3 *upstream region using mVISTA (Fig. 10) revealed a significantly higher number of conserved non-coding sequences (CNS) between mouse and zebrafish than between mouse and *Xenopus *(mouse ↔ zebrafish: 13 CNS, 1325 bp at 52,9%; mouse ↔ *Xenopus*: 1 CNS, 78 bp, 50%), indicating the possibility that *Eya3 *genes gained individual expression patterns and functions among vertebrate species due to evolutionary determined diversity in regulatory mechanisms.

The results of our phenotypic study on *Eya3 *are in line with earlier findings in other mammalian *Eya *genes indicating that their loss does not have an effect on eye development and ocular functions. Eye disorders have not been observed in *Eya1*, *Eya2 *or *Eya4 *mutant mice [14,18,22]. Additionally, histological analysis and detailed investigation of ocular morphology and function by tests such as electroretinography and the optokinetic reflex in the *Eya3 *knockout mutants presented here did not show any differences between the eyes of wild-type controls and hetero- or homozygous *Eya3 *mutant animals, although expression of *Eya3 *was detected in the retina and in the lens epithelium during eye development. In addition to the striking differences between the function of *Eya *genes in *Drosophila *and mice, there are also important differences between the phenotypes of *Eya *mutants among vertebrates. In contrast to morpholino-injected *Xenopus *embryos [23], *Eya3 *mutant mice showed a normal development of neural tissues.

Nevertheless, further investigations revealed clear differences in bone mineral content and body length between wild-type animals and *Eya3 *mutant mice, suggesting an impact of *Eya3 *on physical constitution in general, leading to a reduction of growth. Moreover, *Eya3 *is expressed in the developing muscle, and concomitantly, the forelimb grip strength is reduced in the mutant mice. This might be a reason for the observed decreased locomotor activity of the *Eya3 *mutant mice. Muscle deficiencies have been reported also for *Eya1*^-/-^*Eya2*^-/- ^mutant mice [18], and also for *Six1*, encoding a potential interaction partner of *Eya *proteins, which is also required for proper muscle development [26].

Moreover, the results from additional physiological investigations indicate that *Eya3 *mutant mice suffer also from some heart and lung problems as indicated by some ECG parameters (increased PQ and JT intervals, decreased QRS amplitude) and a decreased tidal volume of the lung. Indeed, *Eya4 *is also involved in cardiac processes as demonstrated previously in human and zebrafish [20]. Taking the lung, heart and muscle alterations together, the significantly reduced locomotion might be understood as a consequence of all these features. Since we have tested the mice only at a young age, these deficits might accumulate during ageing and therefore be responsible for more severe deficits when the mice become older.

To understand the molecular network involving *Eya3*, we analyzed differentially expressed genes in the mutants. We identified several regulated genes in heart and brain indicating regulatory effects of the *Eya3 *mutation or secondary affects based upon missing or changed cell types. Among them, *Nup155 *was confirmed by real-time PCR to be significantly down-regulated in both organs. *Nup155 *is involved in nuclear envelope formation during cell division and is important for the export of macromolecules like mRNAs from the nucleus to the cytoplasm. Depletion of Nup155 leads to a failure of nuclear lamina formation and defects in chromosome segregation at anaphase [27] and therefore has an important impact on cell proliferation. *Nup155 *encodes a protein containing nucleoporin, a non-repetitive/WGA-negative domain. Gene ontology (GO) terms indicate that Nup155 protein is located in a nuclear pore and in a membrane.

Among the 17 down-regulated genes in the heart of *Eya3 *mutant mice, some are associated with specific cardiac functions, e.g. *Tnnc1 *is involved in cardiac muscle contraction, *Calm3 *plays a role in calcium signaling, and *Ankrd1 *is a transcriptional co-factor being required during cardiac myogenesis [28,29].

Furthermore, for several regulated genes in the brain an association with neurodegeneration, particularly with Alzheimer disease has been found. Among the up-regulated genes, *ASS1 *expression levels were significantly higher in glial cells of Alzheimer's disease brains [30]. Among the genes being down-regulated in the brains of *Eya3 *mutants, several are reported to be down-regulated also in Alzheimer's disease, e.g. *ARPP19 *[31], *BIRC4 *[32], *MT3 *[33], *SYT1 *[34] or *TTR *[35]. Moreover, the *SCA10 *gene, which is down-regulated in the brain of the *Eya3 *mutants, is also down-regulated in patients suffering from Parkinson's disease [36]. This short comparison between the differentially expressed genes in the *Eya3 *mutant mice with corresponding features in neurodegenerative disorders in human patients points to an interesting putative role of *EYA3 *in Alzheimer's and Parkinson's disease. This function might be attributed by putative interaction of Eya3 with either Six1 (Six1 binding sites were reported in the *Birc4 *promoter) or with *Pax6 *(Pax6 binding sites have been identified in the *Ass1 *promoter) (data accumulated by Bibliosphere). These functional considerations are based upon associative interpretations of available literature and comparison with the data obtained during the basic characterization of the *Eya3 *mutant opening several interesting avenues for further detailed experimental studies.

## Conclusion

Our study demonstrates that *Eya3 *affects particularly heart, lung and muscle functions. Even if the exploratory behavior is increased, the mutant mice move less than the wild-type mice. Together with the effects on respiratory, muscle and heart function, it might lead to more severe effects, when the mice become older. Therefore, future investigations of *Eya3 *function should focus on effects on aging mice and consider also effects related to neurodegenerative disorders like Alzheimer's or Parkinson's disease.

## Methods

### Generation and molecular characterization of *Eya3 *mutant mice

The *Eya3 *deficient mouse line was generated by insertional mutagenesis in a large-scale gene trap screen (German Gene Trapping Consortium, GGTC). A promoterless reporter gene (*lacZ*) was inserted into the mouse genome by random integration of a gene trap vector (pT1βgeo) [37]. ES cells were derived from strain Sv129S2 (formerly known as Sv129Pasteur). Mutant mice were generated through blastocyst injection and resulting chimeras were bred to C57BL/6 females and crossed to C57BL/6 for four generations. Thus, mice were predominantly C57BL/6. All experiments and housing of the animals were performed according to the German Law on the Protection of Animals (regulation 209.1/211-2531-112/02 by the Government of Upper Bavaria [*Regierung von Oberbayern*]).

For detection of the gene trap vector integration in mutant mice a genomic forward primer was combined with a reverse primer in the *lac*Z cassette of the gene trap vector. Genotyping of mice was performed by Triplex-PCR. A genomic forward primer was either combined with a genomic reverse primer for wild-type amplification or with a reverse primer within the gene trap vector for mutant amplification (table 4). PCR was carried out using the following cycles: 1 × 3 min 94°C; 35 × 94°C 30 sec, 62°C 45 sec, 72°C 60 sec; 1 × 72°C 5 min (PCR-cycler: MJ Research PTC-225, Bio-Rad Laboratories, Munich, Germany).

**Table 4:** PCR primer for characterization of the insertion of the gene trap vector

**Primer**	**Sequence (5' → 3')**
gene trap forward primer (genomic)	TGGGATGCAAACTCGGGTCCTA
gene trap reverse primer (lacZ cassette)	CAAGGCGATTAAGTTGGGTAACG
genotyping forward primer (wild type/mutant)	CAGGAACAGACTGAAACCATGC
genotyping reverse primer (wild type)	AGGTTGTCAGACTGGCACTTCA
genotyping reverse primer (mutant)	CACGCCATACAGTCCTCTTCAC
ex 1_forward primer	CAGAGTGGGTCCGTAATCGT
ex 7_reverse primer	TGCCCAGGATAAGTGGTTTC
ex 8_reverse primer	ATTGCAGATGAAGTGGGTATCAG
ex 8_forward primer	CCAGCCTGATACCCACTTCA
ex 11_reverse primer	ATACCCGTTCCAATTCACTGTC
ex 12_forward primer	CCAACAGTAGTAATTGGCTCAGGT
ex 15_reverse primer	ACCTCGATCTCTGCTCTGAGTCT

### RNA isolation, RT-PCR and Northern blotting

Total RNA was prepared with RNA-Bee (Tel-Test Inc., Friendswood, TX, USA) according to the suppliers protocol. Tissue (50 μg each) was taken from wild-type animals and homozygous mutants from 3 different embryonic stages: E11.5 (whole embryo), E13.5 (head) and E15.5 (head). cDNA was generated by using Superscript II RT (Invitrogen, Karlsruhe, Germany).

Northern Blotting was done with RNA from E15.5 embryos. Samples were initially denatured for 10 min at 68°C and loaded onto an agarose-gel (1,2%, 1 × MOPS-buffer) and separated under denaturing conditions for 4 h (60 V). RNA samples were blotted to Hybond-N^+ ^membrane (Amersham Biosciences, GE Healthcare-Buchler, Braunschweig, Germany) overnight and UV-fixed. Afterwards, the membrane was prehybridized for 2 h with CHURCH-buffer (7% SDS, 50% formamide, 5 × SSC, 2% Roche blocking reagent, 50 mM Na_2_HPO_4_, 0,1% laurylsarcosyl) and then hybridized overnight at 52°C with a digoxigenin-labeled riboprobe (Roche Diagnostics, Mannheim, Germany) against exons 8 – 11 of *Eya3*. Hybridization was followed by washing in 2 × SSC (30 mM Na-Citrat pH 7.0; 300 mM NaCl), 0.1% SDS for 5 min and in 0.1 × SSC, 0.1% SDS for 40 min at 52°C. After incubation with blocking reagent in 100 mM Tris-HCl pH 9.5 and 150 mM NaCl for 30 min at 52°C the membrane was washed in 100 mM Tris-HCl pH 9.5, 150 mM NaCl and incubated with anti-digoxygenin antibody (Roche, diluted 1:5000 in 100 mM Tris-HCl pH 9.5, 150 mM NaCl) for 30 min at room temperature. Finally, the membrane was stained with NBT/BCIP in 100 mM Tris-HCl pH 9.5, 100 mM NaCl, 50 mM MgCl_2 _for 1 h in the dark.

### *LacZ*-staining and *in-situ *hybridization of *Eya3*

*LacZ*-staining was performed on whole mouse embryos of developmental stages E11.5 – E13.5. Mouse embryos were dissected and fixed in 4% paraformaldehyde in PBS. For staining embryos were washed three times in PBS containing 0.01% Na-deoxicholate, 0.02% Non-idet P40, 5 mM EGTA (pH 8.0) and 2 mM MgCl_2 _after fixation. Afterwards, embryos were stained overnight at 37°C in PBS containing 0.5 mg/ml X-gal, 2 mM MgCl_2_, 5 mM K_3_Fe(CN)_6_, 5 mM K_4_Fe(CN)_6_, 0.01% Na desoxycholate, and 0.02% NP-40 in the dark. Before postfixation in 4% paraformaldehyde in PBS at 4°C, several wash steps were carried out in PBS. Embryos were stored in 70% ethanol at 4°C.

*In-situ *hybridizations with paraffin sections were performed to evaluate *Eya3 *expression in the eye. Embryos were fixed in 4% paraformaldehyde in PBS at 4°C, dehydrated and embedded in Jung Histowax (Cambridge Instruments, Nussloch, Germany). Sections (8 μm) were cut with the RM 2065-microtome (Leica, Wetzlar, Germany), mounted onto slices and treated as previously described [38].

### Analysis of zebrafish embryos

Embryos were produced by paired matings of zebrafish (*Danio rerio*, AB strain), and staged in hours post-fertilization (hpf), as described previously [39,40]. *In-situ *hybridization for zebrafish embryos was performed as previously described [41]. For sectioning at the tailbud stage, the stained embryos were cryoprotected in 15% sucrose in phosphate buffer, embedded in 7.5% gelatin, 15% sucrose in PBS, frozen and cryostat-sectioned at 15 μm. For sectioning at the 28 hpf stage, the stained embryos were embedded in 3% agarose and vibratome-sectioned at 70 μm. As probe, we used an *eya3 *cDNA clone generously provided by G. Nica (LMU Munich).

### Phenotyping of mice

A broad phenotypical analysis of 24 wild-type controls (14 ♂, 10 ♀) and 24 *Eya3*^-/- ^mice (14 ♂, 10 ♀) was done in collaboration with the German Mouse Clinic [24]. For histological analysis of the eye 11 week old mice were sacrificed and eyes were removed immediately. Fixation and staining with hematoxylin and eosin were performed using standard-protocols. Funduscopy (age: 11 weeks) and electroretinography (age: 11 weeks, 9 months) were performed as described before [42]. Laser interference biomicroscopy (age: 13 weeks; n = 6) was carried out as previously described [43]. Data from controls and mutant animals were compared using the Student's t-test. Tables show mean value ± standard error of the mean (s.e.m.).

Behavioral phenotyping was done at the age of 8 weeks by using the modified hole board test [44] and carried out as previously described [45]. Data were statistically analyzed using SPSS software (SPSS Inc, Chicago, USA). The forelimb grip strength was tested at the age of 10 weeks with the grip strength meter system, which determines the muscle strength of a mouse. The device exploits the tendency of a mouse to grasp a horizontal metal bar while being pulled by its tail. During the measurement the mouse grasps a special adjustable grip (2 mm) mounted on a force sensor. Five trials were undertaken for each mouse within one minute. Values were presented as means ± standard error of the mean (SEM) and analyzed for the effects of genotype, sex and weight by fitting linear mixed effect models.

To examine the cardiovascular phenotype a surface limb ECG was obtained at the age of 12 – 15 weeks in anesthetized mice. The complete setup was located in a faraday cage. A shape analysis of the ECG traces was performed using the software ECG-auto (EMKA Technologies, Paris, France). For each animal, intervals and amplitudes were measured from five different sets of averaged beats. In addition, the recordings were screened for arrhythmias, including supraventricular and ventricular extrasystoles and conduction blockages. Analysis of variance (ANOVA) was used for multi-factorial analysis of quantitative traits with respect to sex and genotype.

Lung function was determined by whole body plethysmography according to a previously described principle [46]. For measurement a system from Buxco^® ^Electronics (Sharon, Connecticut, USA) was used to assess breathing patterns in unrestrained animals by measuring pressure changes which arise from inspiratory and expiratory temperature and humidity fluctuations during breathing. The system allows transforming pressure swings into flow and volume signals, so that automated data analysis provides tidal volumes, respiratory rates, minute ventilation, inspiratory and expiratory times as well as peak inspiratory and peak expiratory flow rates. Measurements were always performed between 8 a.m. and 11 a.m. to account for potential diurnal variations in breathing.

Gene expression of putative downstream genes were analyzed using Glass cDNA-chips containing the fully sequenced 20K cDNA mouse arrayTAG library (Lion Bioscience, Heidelberg, Germany) [47-50]. A full description of the probes on the microarray is available in the GEO database under GPL4937 [51]. The expression data of *Eya3 *analysis of heart, muscle and brain have been submitted to the GEO database (GSE9999). Total RNA from brain, heart and muscle of 12 – 13 week old male animals (5 controls, 4 mutants) was used for cDNA chip hybridization according to a modified TIGR protocol [52]. Two chip hybridizations including a dye-swap experiment were performed from all selected organs of each individual mutant mouse against the identical pool of the same organ of reference RNAs. Slides were scanned with a GenePix 4000A microarray scanner (GenePix Pro 6.1 image processing software, Axon Instruments, USA). TIGR software package for Microarray analysis [53] including MIDAS (Microarray Data Analysis System) was used for normalization and SAM (Significance Analysis of Microarrays) [54] for the identification of significantly differentially expressed genes. Genes were ranked according to their relative difference value d(i) and selected as significantly regulated with d(i) > 0.5. To estimate the false discovery rate, false positive genes were identified by calculating 1000 permutations of the measurements. The selection of the top differentially expressed genes with reproducible up- or down-regulation includes less than 10% false positives.

The commercial software tool Bibliosphere Pathway Edition (Genomatix Software GmbH, Germany) was used to identify pathways and gene functions that were significantly abundant among the regulated genes. Regulated genes were filtered for two categories of Gene Ontology terms: biological processes and molecular functions. A statistical analysis was performed based on the relative number of observed versus expected annotations of each term. The z-score (> 5.0) indicates whether particular GO annotations are over-represented in a dataset of genes.

Results from Microarray analysis were controlled by real-time PCR for differentially expressed genes in heart and brain with the ABI Prism 7000 Detection System (Applied Biosystems, Darmstadt, Germany). Therefore, the identical total RNA as for the microarray experiments was used. Afterwards, cDNA was prepared with the Ready-to-go T-primed First-strand Kit (Amersham Biosciences, Braunschweig, Germany) according to the manufacturers protocols. Results from real-time PCR were analyzed with the ABI Prism 7000 SDS software and REST (Relative Expression Software Tool [55]).

### Sequence Comparison

For sequence comparison of the 5 kb upstream of the *Eya3 *transcription start sites we used the program mVISTA ([56])

## Abbreviations

ANOVA: analysis of variance; BO: branchio-oto syndrome; BOR: branchio-oto-renal syndrome; ECG: electrocardiography; ED: Eya domain; GGTC: German Gene Trap Consortium; GMC: German Mouse Clinic; GO: gene ontology; PBS: phosphate-buffered saline, RACE: rapid amplification of cDNA ends; RT-PCR: reverse transcription – polymerase chain reaction; SEM: standard error of the mean.

## Authors' contributions

TS, performed the genetic analysis and breeding of the mice, the expression analysis and wrote the manuscript; CD, performed most of the studies of the eyes, analyzed the data and wrote the manuscript; OP, performed some of the studies of the eyes and analyzed the data; TF, created the mouse mutant; LB, performed the neurological screen and analyzed the data; IB, performed the lung function screen and analyzed the data; JF, performed some of the studies at the eyes, analyzed the data and wrote the manuscript; WH, performed the dysmorphology screen and analyzed the data; SMH, performed the behavioral screen, analyzed the data and wrote the manuscript; MH, designed the expression profiling studies and performed the statistical and functional analysis; MK, performed the behavioral screen and analyzed the data; EK, performed the neurological screen and analyzed the data; CM, performed the clinical-chemical screen and analyzed the data; AS, performed the cardio-vascular screen and analyzed the data; CS, designed the experiments on zebrafish and analyzed the data; ST, performed some of the expression studies on zebrafish; VGD, design and coordination of primary workflow and wrote the manuscript; BN, coordination primary workflow; JB, designed the expression profiling and wrote the manuscript; HF, design and coordination of primary workflow, performed the dysmorphology screen, and wrote the manuscript; BI, designed the cardiovascular screen, validated and discussed its results; TK, designed the neurology screen; HS, designed the lung function screen and wrote the manuscript; EW, designed the clinical-chemical screen; WW, designed the gene-trap approach for mouse mutant generation and designed the behavioral screen; LBC, designed the zebrafish experiments, analyzed the data and wrote the manuscript; MHdA, design of concept GMC, primary workflow; study design; JG, designed the experiments, discussed the results at all stages with various partners and wrote the manuscript. All authors read and improved the final manuscript.
